# A Nomogram That Includes Neutrophils and High-Density Lipoprotein Cholesterol Can Predict the Prognosis of Acute Ischaemic Stroke

**DOI:** 10.3389/fneur.2022.827279

**Published:** 2022-02-25

**Authors:** Nan Wang, Hongbing Liu, Mengke Tian, Jing Liang, Wenxian Sun, Luyang Zhang, Lulu Pei, Kai Liu, Shilei Sun, Jun Wu, Yuan Gao, Yuming Xu, Yilong Wang, Bo Song

**Affiliations:** ^1^Department of Neurology, The First Affiliated Hospital of Zhengzhou University, Zhengzhou, China; ^2^Department of Neurology, Beijing Tiantan Hospital, Capital Medical University, Beijing, China

**Keywords:** stroke, neutrophil, nomogram, prognosis, high-density lipoprotein cholesterol

## Abstract

Lipids are implicated in inflammatory responses affecting acute ischaemic stroke prognosis. Therefore, we aimed to develop a predictive model that considers neutrophils and high-density lipoprotein cholesterol to predict its prognosis. This prospective study enrolled patients with acute ischaemic stroke within 24 h of onset between January 2015 and December 2017. The main outcome was a modified Rankin Scale score ≥3 at the 90th day of follow-up. Patients were divided into training and testing sets. The training set was divided into four states according to the median of neutrophils and high-density lipoprotein cholesterol levels in all patients. Through binary logistic regression analysis, the relationship between factors and prognosis was determined. A nomogram based on the results was developed; its predictive value was evaluated through internal and external validations. Altogether, 1,090 patients were enrolled with 872 (80%) and 218 (20%) in the training and testing sets, respectively. In the training set, the major outcomes occurred in 24 (10.4%), 24 (11.6%), 37 (17.2%), and 49 (22.3%) in states 1–4, respectively (*P* = 0.002). Validation of calibration and decision curve analyses showed that the nomogram showed better performance. The internal and external testing set receiver operating characteristics verified the predictive value [area under the curve = 0.794 (0.753–0.834), *P* < 0.001, and area under the curve = 0.973 (0.954–0.992), *P* < 0.001, respectively]. A nomogram that includes neutrophils and high-density lipoprotein cholesterol can predict the prognosis of acute ischaemic stroke, thus providing us with an effective visualization tool.

## Introduction

Stroke is the leading cause of disability and the second leading cause of death worldwide (1). However, stroke is the leading cause of both death and disability in China (2). Ischaemic events are the first to occur in all stroke events, with a rate of ~70% (3). Statistics show that in 2018, 1.57 million stroke deaths were reported in China. Stroke has a significant impact on its socio-economic and family burdens. Therefore, tools that accurately predict its prognosis are needed, especially ischaemic stroke.

Many factors can affect the prognosis of ischaemic stroke, including the immune-inflammatory response, which is an essential process after stroke (4). Neutrophils are the first cells to reach the lesions after stroke and participate in a series of complex inflammatory responses (5). They are involved in the destruction and repair of local brain tissue in cerebral infarction. Studies have shown that inflammatory cells in peripheral blood after stroke also change along with local inflammatory cells (6), thus, testing patients' peripheral blood provides a clinical basis for quick judgment of their prognosis. For example, Zhu et al. (7) have shown that peripheral neutrophil count after stroke is associated with the stroke prognosis.

In addition, lipids are important factors in the inflammatory response. Recent studies have shown that high-density lipoprotein cholesterol (HDL-C) inhibits neutrophil deformation and platelet aggregation (8, 9), and inhibits the formation of neutrophil traps (10). These can prevent further damage to the infarcted tissue after promoting thrombosis. Therefore, we aimed to develop a predictive model that includes neutrophils and high-density lipoprotein cholesterol to predict the prognosis of acute ischaemic stroke (AIS).

## Subjects and Methods

### Study Population

This was a prospective consecutive hospital-based cohort study (11, 12). The AIS diagnosis was based on the criteria defined by the World Health Organization (13). Our study was approved by the Ethics Committee, and written informed consent was obtained from all participating patients and their families. We recruited patients with AIS who were admitted from January 2015 to December 2017.

The inclusion criteria were AIS patients admitted within 24 h of stroke onset in the database (not include patients with thrombectomy and admission with any infection). The exclusion criteria were as follows: patients who lacked blood cell count or lipid data; Immune diseases/chronic inflammatory diseases; patients with cancer or haematologic diseases or were using immunosuppressants; patients who had major trauma or surgery; patients with severe liver or kidney disease or chronic lung disease; and patients with incomplete follow-up information.

### Data Collection

Baseline information was obtained from trained professionals who collected patient admission reports. Demographic characteristics included sex, age, and personal history (e.g., smoking). Clinical data included history and degree of stroke according to the National Institutes of Health Stroke Scale (NIHSS) at the time of admission. Laboratory tests included routine blood and lipid analyses obtained from the patients within 24 h of AIS onset. An automated analyser (Coulter LH 750 Hematology Analyzer; Beckman Coulter, Brea, CA, USA) was used to analyse the blood cell counts. All serum biochemical parameters were measured using a fully automatic biochemical analyser (COBAS 8000 fully automatic biochemical analyser; Roche, Basel, Switzerland). The routine blood cell count included white blood cells and neutrophils. Lipid analysis included the total cholesterol, triglycerides, HDL-C, and low-density lipoprotein.

### Follow-Up and Assessment

Each patient completed 90 days of face-to-face or telephone follow-ups. The patients were assessed using the modified Rankin Scale (mRS) scale during follow-up. An mRS score ≥3 was defined as a poor prognosis, with mRS = 6 points representing death. An mRS score <3 was classified as a better prognosis. The main outcome of interest in this study was poor prognosis in patients during the 90-day follow-up.

### Grouping and Design

The patients were grouped based on the admission time. The first four sessions were selected as the training set, and the last session was the testing set (with a 4:1 ratio), and the training set was compared with the testing set. The training set is used to fit the predictive model and develop the nomogram, and the testing set to validate the model externally. First, we determined the median of neutrophils and HDL-C levels in study populations and divided them into high and low groups. Afterwards, four combined neutrophils and high-density lipoprotein cholesterol states were formed: state 1, group with low neutrophils and high HDL-C; state 2, group with low neutrophils and low HDL-C; state 3, group with high neutrophils and high HDL-C; state 4, group with high neutrophils and low HDL-C. The different states were compared for poor outcomes on the 90th day of follow-up. A univariate logistic regression analysis was conducted, and variables showing significance were included in a multivariate logistic regression analysis to develop a nomogram that predicts the 90-day prognosis. Internal and external evaluations were carried out in the training and testing sets, respectively. The evaluation methods included calibration curve, decision curve analysis, and receiver operating characteristic (ROC) curve.

### Statistical Analyses

All data analyses for this study were performed using SPSS Statistics version 23.0 (IBM Corp., Armonk, NY, USA). In the training set, the classification data were expressed in frequency (%), and inter-group comparison was performed using either the χ^2^ test or the Fisher precision test. The relationship between the variables and 90-day prognosis was analyzed using logistic regression. Nomogram development and performance evaluation were performed using R Studio based on R x64 4.0.4 (The R Project, Vienna, Austria). A two-sided *P*-value of <0.05 indicate significant difference and statistical significance.

## Results

### Comparison of the Training Set With the Testing Set

**Figure 1:**
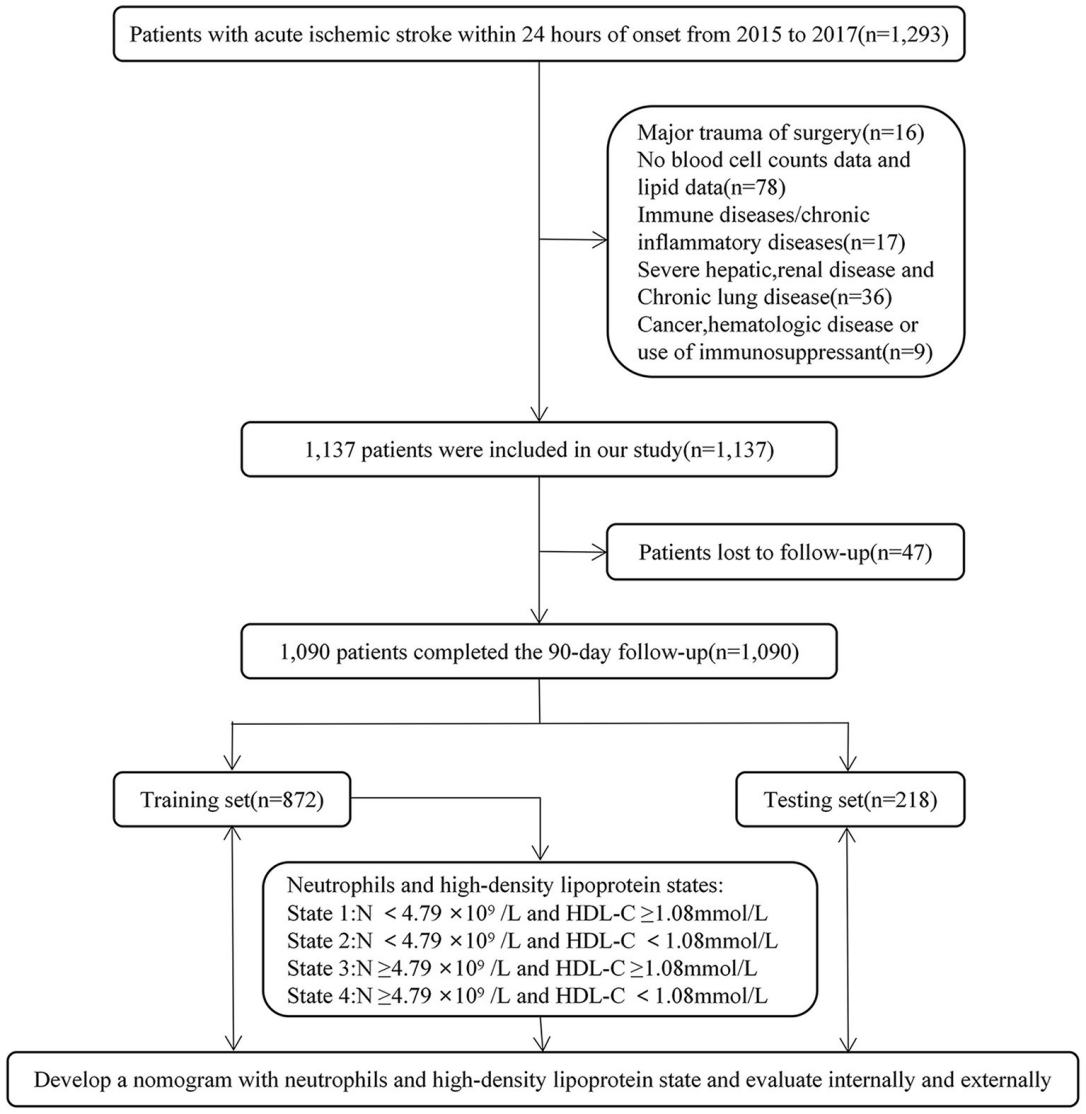
Research process and design. N, Neutrophil; HDL-C, high-density lipoprotein cholesterol.

According to the research process (Figure 1), 1,090 patients met our study criteria, men, *n* = 738, 67.7%; age ≥60 years, *n* = 600, 55.0%; with major outcome, *n* = 165, 15.1%. The characteristics of the patients and the comparison of the training and testing sets are shown in Table 1. There were no significant differences in most variables in the group comparison between the training and testing sets, indicating high similarity between the sets. Therefore, the models fitted by the training set will be well-representative.

**Table 1:** Comparison of training set and testing set.

**Variable**	**Total**	**Training set**	**Testing set**	***P*** **-value**
	***N*** **= 1,090**	***n*** **= 872**	***n*** **= 218**	
Age ≥60 y, n (%)	600 (55.0%)	478 (54.8%)	122 (56.0%)	0.761
Male, *n* (%)	738 (67.7%)	588 (67.4%)	150 (68.8%)	0.698
Smoking, *n* (%)	382 (35.0%)	290 (33.3%)	92 (42.2%)	0.013
NIHSS >5 on admission, *n* (%)	313 (28.8%)	261 (30.0%)	52 (24.2%)	0.092
mRS score >1 before stroke, *n* (%)	23 (2.1%)	17 (1.9%)	6 (2.8%)	0.435
**Medical history**, ***n*** **(%)**				
Stroke	243 (22.3%)	190 (21.8%)	53 (24.3%)	0.423
Coronary heart diseases	128 (11.7%)	98 (11.2%)	30 (13.8%)	0.301
Atrial fibrillation	60 (5.5%)	52 (6.0%)	8 (3.7%)	0.184
Hypertension	648 (59.4%)	519 (59.5%)	129 (59.2%)	0.926
Diabetes mellitus	225 (20.7%)	171 (19.6%)	54 (24.8%)	0.094
Dyslipidemia	114 (10.5%)	99 (11.4%)	15 (6.9%)	0.053
**Laboratory examination**				
White blood cell >9.5 × 10^9^ /L, *n* (%)	171 (15.7%)	136 (15.6%)	35 (16.1%)	0.868
Neutrophil >6.3 × 10^9^ /L, *n* (%)	214 (19.6%)	168 (19.3%)	46 (21.1%)	0.542
TC ≥5.2 mmol/L, *n* (%)	191 (17.7%)	164 (19.1%)	27 (12.4%)	0.021
LDL-C ≥3.61 mmol/L, *n* (%)	158 (14.7%)	135 (15.8%)	23 (10.6%)	0.053
HDL-C ≤ 0.91 mmol/L, *n* (%)	252 (23.1%)	196 (22.5%)	56 (25.7%)	0.315
**Short-term outcome**, ***n*** **(%)**				
Poor outcome (mRS >2)	165 (15.1%)	134 (15.4%)	31 (14.2%)	0.673

*NIHSS, national institute of health stroke scale; mRS, modified rankin scale; TC, total cholesterol; LDL-C, low density lipoprotein-cholesterol; HDL-C, high-density lipoprotein cholesterol*.

### Baseline Characteristics of the Training Set

In the 872 patients in the training set, men, *n* = 588, 67.4%; age ≥60 years, *n* = 394, 45.2%; with major outcome, *n* = 134, 15.4%; states 1, 2, 3, and 4 had 230, 207, 215, and 220 patients, respectively (Table 2). The proportion of patients with poor prognosis was increasing in the 90-day follow-up, 24 (10.4%), 24 (11.6%), 37 (17.2%), and 49 (22.3%) in states 1–4, respectively (*P* = 0.002).

**Table 2:** Baseline characteristics of the training set.

**Variable**	**Total**	**State 1**	**State 2**	**State 3**	**State 4**	***P*** **-value**
	***N*** **= 872**	***n*** **= 230**	***n*** **= 207**	***n*** **= 215**	***n*** **= 220**	
Age ≥60 y, *n* (%)	394 (45.2%)	79 (34.3%)	100 (48.3%)	97 (45.1%)	118 (53.6%)	<0.001
Male, *n* (%)	588 (67.4%)	131 (57.0%)	148 (71.5%)	134 (62.3%)	175 (79.5%)	<0.001
Smoking, *n* (%)	290 (33.3%)	65 (28.3%)	73 (35.3%)	58 (27.0%)	94 (42.7%)	0.001
NIHSS >5 on admission, *n* (%)	261 (30.0%)	50 (21.8%)	56 (27.1%)	65 (30.4%)	90 (40.9%)	<0.001
mRS score >1 before stroke, *n* (%)	17 (1.9%)	4 (1.7%)	6 (2.9%)	2 (0.9%)	5 (2.3%)	0.509
**Medical history**, ***n*** **(%)**						
Stroke	190 (21.8%)	58 (25.2%)	44 (21.3%)	45 (20.9%)	43 (19.5%)	0.500
Coronary heart diseases	98 (11.2%)	25 (10.9%)	20 (9.7%)	24 (11.2%)	29 (13.2%)	0.710
Atrial fibrillation	52 (6.0%)	17 (7.4%)	7 (3.5%)	15 (7.0%)	13 (5.9%)	0.297
Hypertension	519 (59.5%)	138 (60.0%)	118 (57.0%)	123 (57.2%)	140 (63.6%)	0.459
Diabetes mellitus	171 (19.6%)	33 (14.3%)	43 (20.4%)	43 (20.0%)	53 (24.1%)	0.074
Dyslipidemia	99 (11.4%)	18 (7.8%)	26 (12.6%)	22 (10.2%)	33 (15.0%)	0.096
**Laboratory examination**						
White blood cell >9.5 × 10^9^ /L, *n* (%)	136 (15.6%)	0 (0.0%)	0 (0.0%)	72 (33.5%)	64 (29.1%)	<0.001
TC ≥5.2 mmol/L, *n* (%)	164 (19.1%)	58 (25.3%)	27 (13.2%)	46 (21.8%)	33 (15.3%)	0.004
LDL-C ≥3.61 mmol/L, *n* (%)	135 (15.8%)	41 (18.1%)	23 (11.2%)	39 (18.6%)	32 (14.9%)	0.141
**Short-term outcome**, ***n*** **(%)**						
Poor outcome (mRS >2)	134 (15.4%)	24 (10.4%)	24 (11.6%)	37 (17.2%)	49 (22.3%)	0.002

*NIHSS, national institute of health stroke scale; mRS, modified rankin scale; TC, total cholesterol; LDL-C, low density lipoprotein-cholesterol; Neutrophils and high-density lipoprotein cholesterol states: State 1: N <4.79 × 10^9^ /L and HDL-C ≥1.08 mmol/L; State 2: N <4.79 × 10^9^ /L and HDL-C <1.08 mmol/L; State 3: N ≥4.79 × 10^9^ /L and HDL-C ≥1.08 mmol/L; State 4: N ≥4.79 × 10^9^ /L and HDL-C <1.08 mmol/L; N, Neutrophil; HDL-C, high-density lipoprotein cholesterol*.

### Neutrophils and High-Density Lipoprotein Cholesterol States With Prognosis

We first conducted a univariate logistic regression analysis to explore the factors that affect poor prognosis during the 90-day follow-up (Table 3). There are significant differences in those aged ≥60 years, men, those with NIHSS of >5 on admission, mRS score >1 before stroke, those with stroke, coronary heart diseases, and atrial fibrillation, and states 3 and 4. The multivariate logistic regression analysis (Table 3) revealed that age ≥60 years [odds ratio (OR) = 1.887, 95% confidence interval (95% CI): 1.205–2.955, *P* = 0.006], NIHSS >5 on admission (OR = 7.200, 95% CI: 4.678–11.081, *P* < 0.001), mRS score >1 before stroke (OR = 4.945, 95% CI: 1.593–15.348, *P* = 0.006), stroke (OR = 1.866, 95% CI: 1.155–3.015, *P* = 0.011), and state 4 (OR = 2.224, 95% CI: 1.215–4.072, *P* = 0.010) were significantly different.

**Table 3:** Logistic regression analysis of different variables and prognosis in training set.

**Variables**	**Univariate analysis**		**Multivariate analysis**	
	**OR (95% CI)**	***P*** **-value**	**OR (95% CI)**	***P*** **-value**
Age ≥60y, *n* (%)	1.845 (1.251–2.721)	0.002	1.887 (1.205–2.955)	0.006
Male, *n* (%)	0.600 (0.412–0.874)	0.008	0.687 (0.440–1.071)	0.098
Smoking, *n* (%)	0.731 (0.486–1.100)	0.133		
NIHSS >5 on admission, *n* (%)	7.542 (5.024–11.320)	<0.001	7.200 (4.678–11.081)	<0.001
mRS score >1 before stroke, *n* (%)	8.422 (3.147–22.539)	<0.001	4.945 (1.593–15.348)	0.006
Stroke, *n* (%)	1.820 (1.211–2.734)	0.004	1.866 (1.155–3.015)	0.011
Coronary heart diseases, *n* (%)	2.089 (1.271–3.435)	0.004	1.448 (0.797–2.630)	0.225
Atrial fibrillation, *n* (%)	3.530 (1.941–6.418)	<0.001	1.853 (0.878–3.910)	0.106
Hypertension, *n* (%)	1.417 (0.962–2.087)	0.078		
Diabetes mellitus, *n* (%)	1.418 (0.919–2.190)	0.115		
Dyslipidemia, *n* (%)	0.895 (0.492–1.627)	0.895		
White blood cell >9.5 × 10^9^ /L, *n* (%)	1.146 (0.701–1.874)	0.587		
TC ≥5.2 mmol/L, *n* (%)	1.185 (0.750–1.873)	0.466		
LDL–C ≥3.61 mmol/L, *n* (%)	1.257 (0.772–2.045)	0.358		
State 1	1 (reference)		1 (reference)	
State 2	1.126 (0.618–2.051)	0.699	1.132 (0.581–2.207)	0.715
State 3	1.784 (1.028–3.097)	0.040	1.773 (0.963–3.266)	0.066
State 4	2.460 (1.450–4.173)	0.001	2.224 (1.215–4.072)	0.010

*NIHSS, national institute of health stroke scale; mRS, modified rankin scale; TC, total cholesterol; LDL-C, low density lipoprotein-cholesterol; Neutrophils and high-density lipoprotein cholesterol states: State 1: N <4.79 × 10^9^ /L and HDL-C ≥1.08 mmol/L; State 2: N <4.79 × 10^9^ /L and HDL-C <1.08 mmol/L; State 3: N ≥4.79 × 10^9^ /L and HDL-C ≥1.08 mmol/L; State 4: N ≥4.79 × 10^9^ /L and HDL-C <1.08 mmol/L; N, Neutrophil; HDL-C, high-density lipoprotein cholesterol; Multi-factor logistic regression analysis included: age ≥60 y, male, NIHSS>5 on admission, mRS score>1, history of stroke, coronary heart diseases, atrial fibrillation, neutrophils and high-density lipoprotein cholesterol states*.

### Development and Evaluation of Nomogram

**Figure 2:**
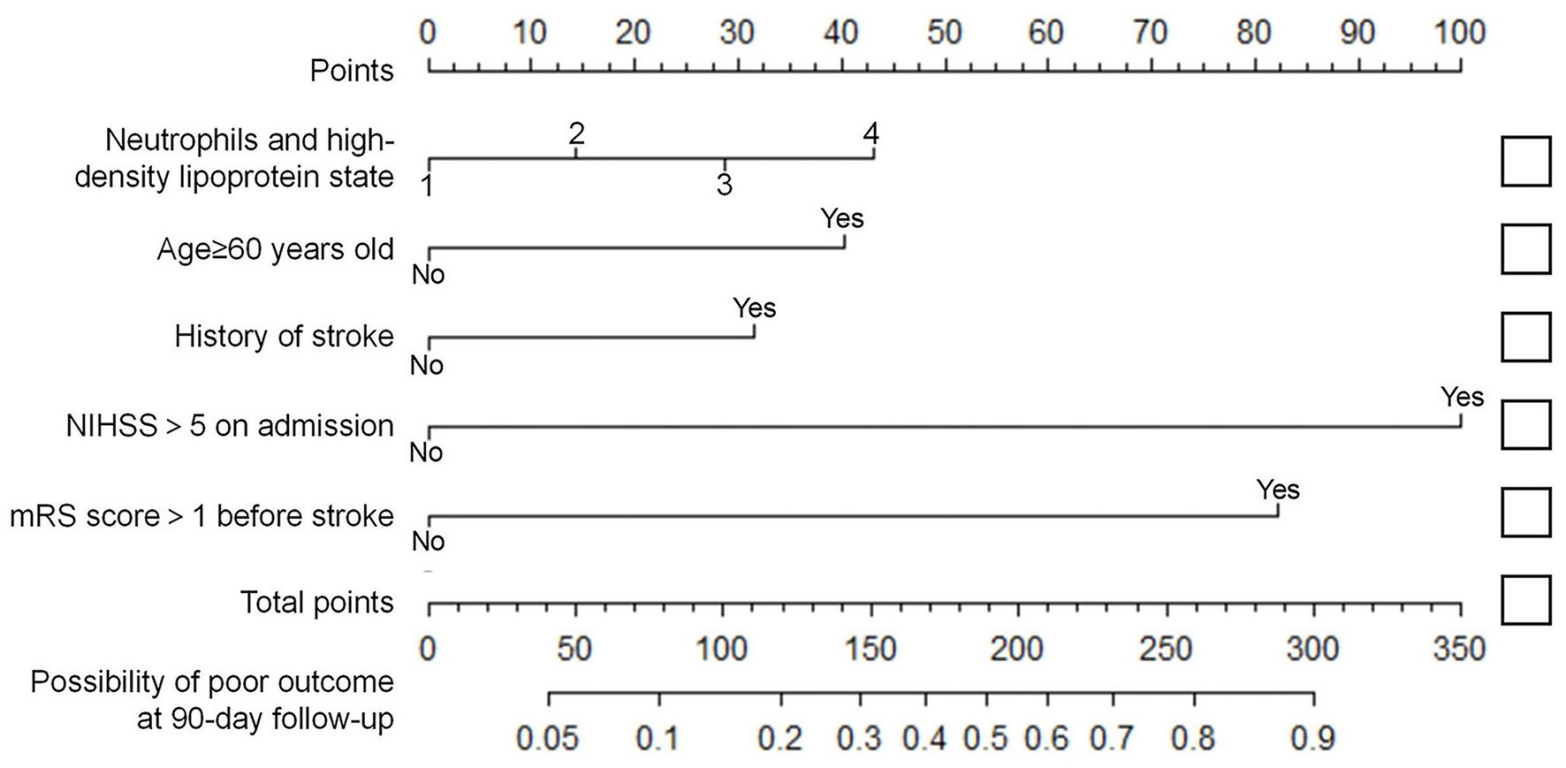
Nomogram for predicting short-term prognosis of acute ischemic stroke. In the neutrophils and high-density lipoprotein cholesterol states variable, state 1 scored 0 points, state 2 scored 14.3 points, state 3 scored 28.7 points, and state 4 scored 43.0 points. Age ≥60 years old, with a history of stroke, mRS >1 before the onset and NIHSS >5 at admission were recorded as 40.3 points, 31.5 points, 82.2 points and 100 points, respectively. Otherwise score 0 points (see Supplementary Materials). State 1: N <4.79 × 10^9^ /L and HDL-C ≥1.08 mmol/L; State 2: N <4.79 × 10^9^ /L and HDL-C <1.08 mmol/L; State 3: N ≥4.79 × 10^9^ /L and HDL-C ≥1.08 mmol/L; State 4: N ≥4.79 × 10^9^ /L and HDL-C <1.08 mmol/L; N, Neutrophil; HDL-C, high-density lipoprotein cholesterol.

**Figure 3:**
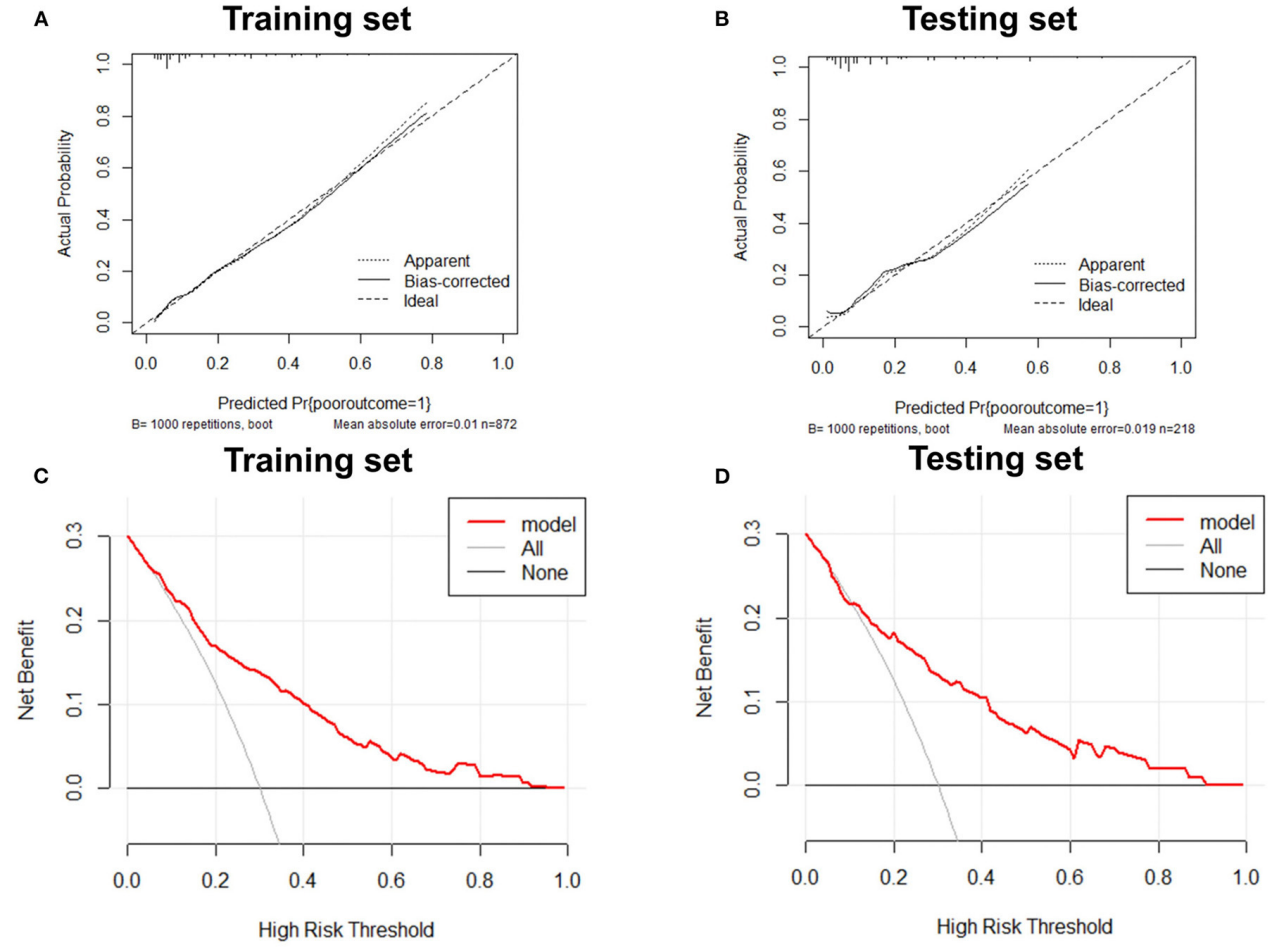
Calibration curve and decision curve analysis of training set and testing set. Internal and external validation of predictive models. **(A)** The calibration curve of the prediction model in the training set; **(B)** The calibration curve of the prediction model in the testing set; **(C)** The decision curve analysis of the prediction model in the training set; **(D)** The decision curve analysis of the prediction model in the testing set.

**Figure 4:**
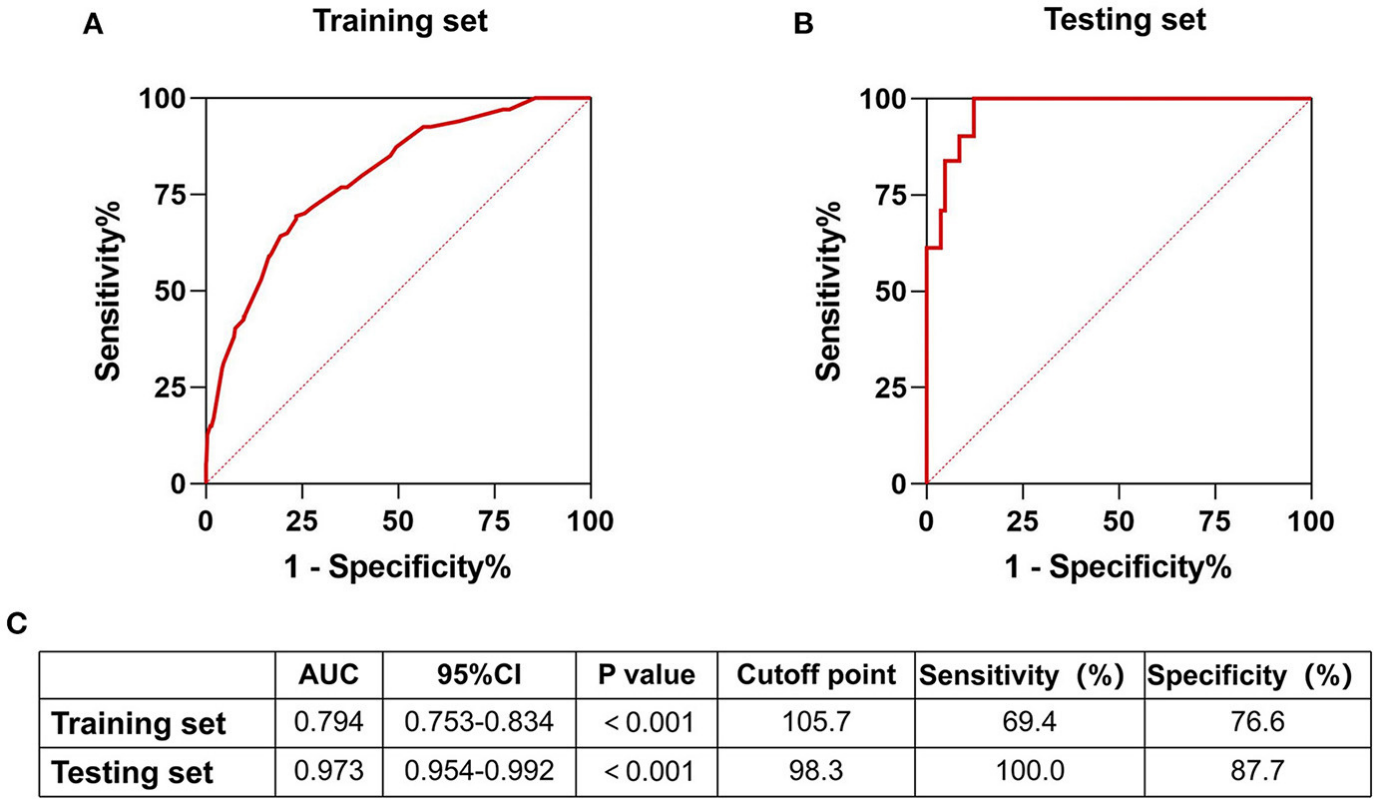
Receiver operating curve of training set and testing set. Internal and external validation of predictive models. **(A)** The receiver operating curve of the prediction model in the training set; **(B)** The receiver operating curve of the prediction model in the testing set; **(C)** Part of the result data display of the receiver operating curve of the nomograph in the training set and the test set.

Variables with significant differences in the multivariate logistic regression analysis, including age ≥60 years, NIHSS >5 on admission, mRS score >1 before stroke, history of stroke, neutrophils, and high-density lipoprotein cholesterol states, were selected for the development of the nomogram (Figure 2). We conducted an internal effect evaluation by the training set for the nomogram. The calibration and decision curve analyses showed good consistency and practicability of the model (Figures 3A,C), and the ROC curve showed a good predictive effect [area under the curve = 0.794 (0.753–0.834), *P* < 0.001] (Figures 4A,C). To further confirm the accuracy of the nomogram, we conducted an external effect evaluation by the testing set that had nothing to do with the construction of the model. Similarly, the calibration curve and decision curve analysis showed good consistency and practicability of the model (Figures 3B,D), and the ROC curve showed a good prediction effect [area under the curve = 0.973 (0.954–0.992), *P* < 0.001] (Figures 4B,C).

## Discussion

Inflammation is a new prevention target in stroke risk, and the secondary prevention of vascular risk after stroke requires new treatment methods (14). Thus, new indicators and markers as early warning signs are needed. Most predictive models currently include only traditional risk factors. Hence, the development of predictive models with inflammation and lipids is needed. Recently, Li et al. (15) examined the effects of residual inflammation risk on stroke and transient ischaemic attack (TIA) prognosis and found that those with high cholesterol levels and high-sensitivity C-reactive protein had a worse prognosis than those with only high levels of high-sensitivity C-reactive protein. Based on these ideas, the correlation of neutrophils and HDL-C states with prognosis of AIS was analyzed, and a clinically practical nomogram was developed. The results showed that the proportion of poor outcomes in the four states increased, proving the difference in prognosis in various states. The multivariate analysis revealed a significant difference and independent correlation between state 4 and poor outcome at the 90-day follow-up (OR = 2.224, 95% CI: 1.215–4.072, *P* = 0.010). Our nomogram, which includes neutrophils and HDL-C states, has been developed to provide clinicians and patients with visual and practical predictive tools. The evaluation of the effect for the nomogram by internal validation in training set and external validation in testing set fully demonstrated the advantages of the model.

Studies have shown a close link between inflammation and lipid media (16, 17). Few previous studies that have used the combination of inflammation and lipids to predict the prognosis of AIS have recently attracted increasing attention from scholars worldwide. For example, Yin and Kou et al. found that the neutrophil-to-HDL ratio is not only closely related to coronary artery stenosis but is also an independent predictor of severe coronary artery stenosis (18). Based on this ratio, it will be interesting to explore the relationship between the different states of neutrophils and HDL-C composition and stroke prognosis.

Numerous studies have shown that neutrophils are closely related to the prognosis of stroke. The involvement of neutrophils after stroke was confirmed in experimental animals and tissues from stroke patients who died (19). Neutrophils are involved in the acute and recovery period; thus, their effect on the AIS prognosis is significant (20). These indicate that neutrophils are involved in the infarct tissue in space and time. On a deeper level, a study has shown that the formation of neutrophil traps promotes thrombosis and further destroys lesions (21). Meanwhile, after stroke, neutrophils can bind to adhesive molecules in the inner walls of blood vessels and remain in the capillaries around the lesions (22). The above studies show that neutrophils can greatly affect functional recovery. On the other hand, Zhu et al. (7) confirmed the prognostic relationship between neutrophils in peripheral blood and AIS and TIA. This shows that although we cannot directly detect neutrophils in the infarcted area, the neutrophil count in the peripheral blood can still provide us with a meaningful reference. What's more, HDL-C, a lipid, is a recognized vascular protection factor (23). A meta-analysis of prospective cohort studies showed that high HDL-C levels reduced the incidence of stroke (24), while low HDL-C levels were associated with higher stroke severity and poor clinical prognosis (25). Studies have shown that HDL-C regulates the activity of a variety of inflammatory cells, including neutrophils and eosinophils (26). Further studies have shown that HDL-C can inhibit neutrophil activity and transformation (10). The effect of HDL-C inhibiting neutrophils is the basis of our research. We hope to see the prognosis of AIS in different neutrophils and high-density lipoprotein cholesterol states.

From a researcher's point of view, the results suggest that interventions against neutrophils and HDL-C may be potential therapeutic targets for improving the prognosis of AIS. Neutrophil retention still occurs in the capillaries after AIS revascularization, reducing blood supply to the end brain tissue. This is one of the important factors affecting the prognosis of stroke. Studies have shown that injections to remove neutrophil reagents improve stroke prognosis (27). Studies have also shown that HDL-C confers endotheliocyte protection. Using an experimental model of AIS, the researchers demonstrated the role of HDL-C receptor scavenger receptor class B type I, which is expressed by endothelial cells, in the neuroprotective effect of HDL-C. The results show that stroke volume can be reduced to improve prognosis (28). The above studies suggest that interventions for neutrophils and HDL-C and improve the prognosis of stroke still need a lot of work.

This study had some limitations. First, the sample size was relatively small and was from a single hospital, hence, there may be selection bias. Second, when the patients were grouped into the training and testing sets, they were divided according to admission time. There may be selection bias, because of the differences in medical and health conditions before and after admission time. Finally, we studied the blood from the peripheral veins of patients with ischaemic stroke who were admitted within 24 h without considering the dynamic changes in inflammatory cells and lipids in peripheral blood over time. Nevertheless, neutrophils and HDL-C states can be used as predictive indicators for the AIS prognosis, and a nomogram has been developed for clinical prediction, although it still needs to be expanded further for larger-scale verification in more centers and with larger sample sizes.

In conclusion, the study found that neutrophils and HDL-C can be used to predict the patients' prognosis 90 days after AIS. The developed nomogram, which includes neutrophils and high-density lipoprotein cholesterol, can provide clinicians and patients with visual prediction of the prognosis of AIS.

## Data Availability Statement

The raw data supporting the conclusions of this article will be made available by the authors, without undue reservation.

## Ethics Statement

The studies involving human participants were reviewed and approved by the Ethics Committee of the First Affiliated Hospital of Zhengzhou University. The patients/participants provided their written informed consent to participate in this study.

## Author Contributions

BS and YX designed the overall study. NW designed and carried out experiments and collected data by NW, HL, MT, JL, LZ, WS, LP, and KL and so on. NW wrote the manuscript. BS and YW supervised this study, designed experiments, and edited the article. All authors contributed to the article and approved the submitted version.

## Funding

This study was funded by the National Key Research and Development Program, Major Chronic Non-communicable Disease Prevention and Control Research Key Special Project (2017YFC1308202) and the Non-profit Central Research Institute Fund of Chinese Academy of Medical Sciences (2020-PT310-01).

## Conflict of Interest

The authors declare that the research was conducted in the absence of any commercial or financial relationships that could be construed as a potential conflict of interest.

## Publisher's Note

All claims expressed in this article are solely those of the authors and do not necessarily represent those of their affiliated organizations, or those of the publisher, the editors and the reviewers. Any product that may be evaluated in this article, or claim that may be made by its manufacturer, is not guaranteed or endorsed by the publisher.
