# Supplementary material for: A Nomogram That Includes Neutrophils and High-Density Lipoprotein Cholesterol Can Predict the Prognosis of Acute Ischaemic Stroke
Source: Front Neurol. 2022 Feb 25;13:827279. doi: 10.3389/fneur.2022.827279 (PMC8914087; doi:10.3389/fneur.2022.827279)
Supplement: Supplementary file 1 [file Data_Sheet_1.pdf]

Supplementary material in **FIGURE 2：**

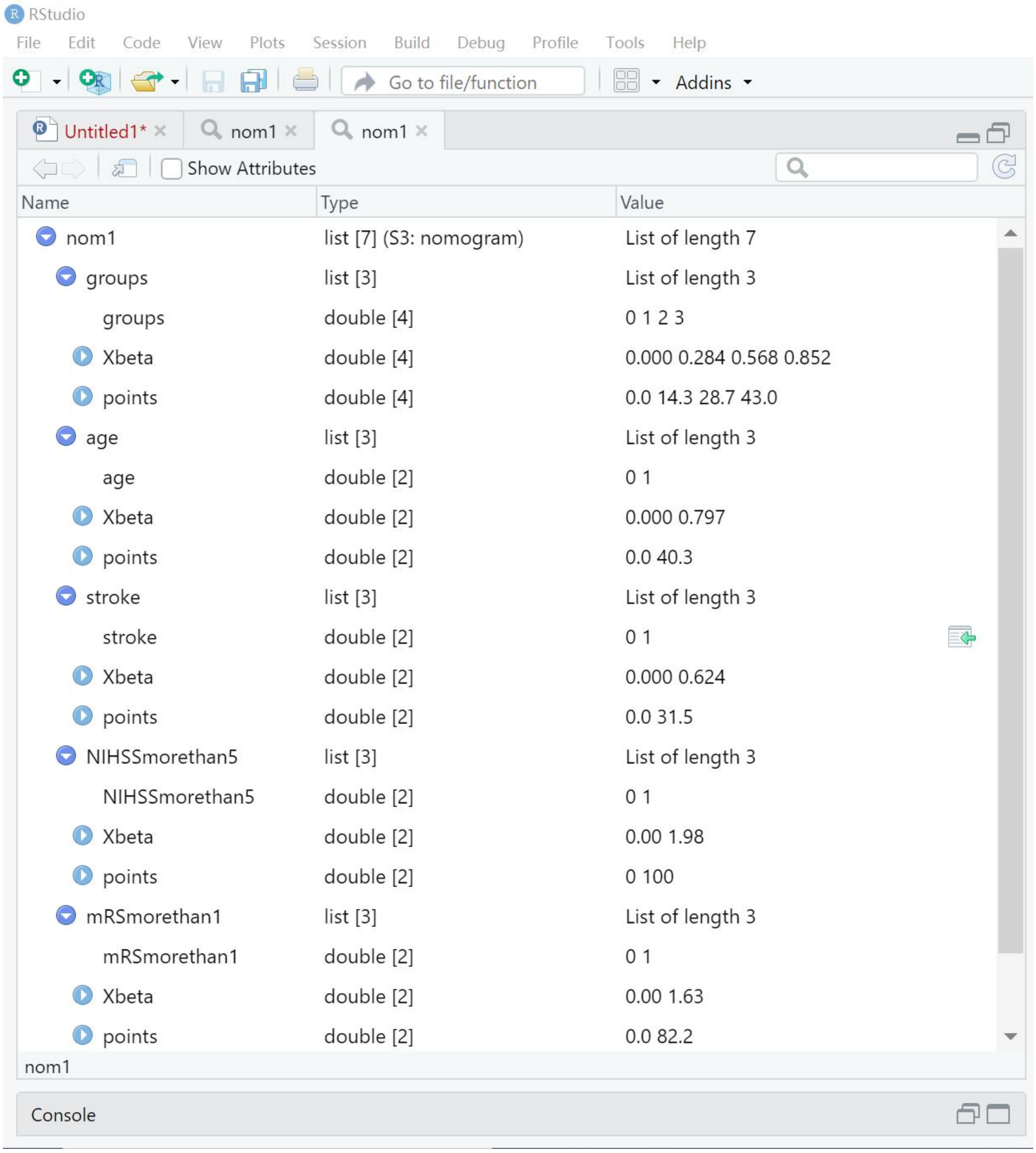

Name	Type	Value
nom1	list [7] (S3: nomogram)	List of length 7
groups	list [3]	List of length 3
groups	double [4]	0 1 2 3
Xbeta	double [4]	0.000 0.284 0.568 0.852
points	double [4]	0.0 14.3 28.7 43.0
age	list [3]	List of length 3
age	double [2]	0 1
Xbeta	double [2]	0.000 0.797
points	double [2]	0.0 40.3
stroke	list [3]	List of length 3
stroke	double [2]	0 1
Xbeta	double [2]	0.000 0.624
points	double [2]	0.0 31.5
NIHSSmorethan5	list [3]	List of length 3
NIHSSmorethan5	double [2]	0 1
Xbeta	double [2]	0.00 1.98
points	double [2]	0 100
mRSmorethan1	list [3]	List of length 3
mRSmorethan1	double [2]	0 1
Xbeta	double [2]	0.00 1.63
points	double [2]	0.0 82.2

Scores for different groups based on R's nomogram.
groups= neutrophils and high-density lipoprotein states
age= Age⩾60 years old
stroke= with a history of stroke
NIHSSmorethan5= NIHSS> 5 at admission
mRSmorethan1= mRS > 1 before the onset
